# High-risk patients with locally advanced non-small cell lung cancer treated with stereotactic body radiation therapy to the peripheral primary combined with conventionally fractionated volumetric arc therapy to the mediastinal lymph nodes

**DOI:** 10.3389/fonc.2022.1035370

**Published:** 2023-01-13

**Authors:** Tanja Eichkorn, Jonathan W. Lischalk, Cedric Stüwe, Eric Tonndorf-Martini, Kai Schubert, Lisa-Antonia Dinges, Sebastian Regnery, Farastuk Bozorgmehr, Laila König, Petros Christopoulos, Juliane Hörner-Rieber, Sebastian Adeberg, Klaus Herfarth, Hauke Winter, Michael Thomas, Stefan Rieken, Jürgen Debus, Rami A. El Shafie

**Affiliations:** ^1^ Department of Radiation Oncology, Heidelberg University Hospital, Heidelberg, Germany; ^2^ National Center for Radiation Oncology (NCRO), Heidelberg Institute for Radiation Oncology (HIRO), Heidelberg, Germany; ^3^ National Center for Tumor diseases (NCT) Heidelberg University Hospital, Heidelberg, Germany; ^4^ Department of Radiation Oncology, Perlmutter Cancer Center, New York University Langone Health at Long Island, New York, NY, United States; ^5^ Thoracic Clinic, Heidelberg University, Heidelberg, Germany; ^6^ Translational Lung Research Center (TLRC), Member of German Center for Lung Research Deutsches Zentrum für Lungenforschung (DZL), Heidelberg, Germany; ^7^ Department of Thoracic Surgery, Thoracic Clinic, Heidelberg University, Heidelberg, Germany; ^8^ Department of Radiation Oncology, Göttingen University Hospital, Göttingen, Germany; ^9^ Clinical Cooperation Unit Radiation Oncology (E050), German Cancer Research Center (dkfz), Heidelberg, Germany; ^10^ Deutsches Konsortium für Translationale Krebsforschung (DKTK), Partner Site Heidelberg, German Cancer Research Center (dkfz), Heidelberg, Germany

**Keywords:** locally advanced non-small cell lung cancer (NSCLC), peripherally located NSCLC, radiation therapy, dosimetric comparison, pulmonary toxicity, high-risk patients

## Abstract

**Introduction:**

A very narrow therapeutic window exists when delivering curative chemoradiotherapy for inoperable locally advanced non-small cell lung cancer (NSCLC), particularly when large distances exist between areas of gross disease in the thorax. In the present study, we hypothesize that a novel technique of stereotactic body radiation therapy (SBRT) to the primary tumor in combination with volumetric arc therapy (VMAT) to the mediastinal lymph nodes (MLN) is a suitable approach for high-risk patients with large volume geographically distant locally advanced NSCLC.

**Patients and methods:**

In this single institutional review, we identified high-risk patients treated between 2014 and 2017 with SBRT to the parenchymal lung primary as well as VMAT to the involved MLN using conventional fractionation. Dosimetrically, comparative plans utilizing VMAT conventionally fractionated delivered to both the primary and MLN were analyzed. Clinically, toxicity (CTCAE version 5.0) and oncologic outcomes were analyzed in detail.

**Results:**

A total of 21 patients were identified, 86% (n=18) of which received chemotherapy as a portion of their treatment. As treatment phase was between 2014 and 2017, none of the patients received consolidation immunotherapy. Target volume (PTV) dose coverage (99 vs. 87%) and CTV volume (307 vs. 441 ml) were significantly improved with SBRT+MLN vs. for VMAT alone (p<0.0001). Moreover, low-dose lung (median V5Gy [%]: 71 vs. 77, p<0.0001), heart (median V5Gy [%]: 41 vs. 49, p<0.0001) and esophagus (median V30Gy [%]: 54 vs. 55, p=0.03) dose exposure were all significantly reduced with SBRT+MLN. In contrast, there was no difference observed in high-dose exposure of lungs, heart, and spinal cord. Following SBRT+MLN treatment, we identified only one case of high-grade pneumonitis. As expected, we observed a higher rate of esophagitis with a total of seven patients experience grade 2+ toxicity. Overall, there were no grade 4+ toxicities identified. After a median 3 years follow up, disease progression was observed in 70% of patients irradiated using SBRT+MLN, but never in the spared ‘bridging’ tissue between pulmonary SBRT and mediastinal VMAT.

**Conclusion:**

For high risk patients, SBRT+MLN is dosimetrically feasible and can provide an alternative to dose reductions necessitated by otherwise very large target volumes.

## 1 Introduction

Locally advanced non-small cell lung cancer (NSCLC) is a very challenging disease site to treat definitively given the patient population often times has significant comorbidities as a consequence of chronic tobacco abuse leading to cardiovascular and pulmonary disease. Moreover, definitive chemoradiation therapy in and of itself is a very difficult treatment to complete for patients and carries relatively high risks of esophagitis and pneumonitis. The very narrow therapeutic window in this disease site has been demonstrated in the RTOG 0617 dose escalation trial. Follow-up analysis of this dose escalation trial demonstrated radiation doses to the heart were associated with survival endpoints, and radiation dose to the lungs was associated with high-grade toxicity. Moreover, advanced radiation techniques, specifically intensity-modulated radiotherapy (IMRT), was shown to mitigate treatment-related toxicity (1). As such, implementation of novel radiotherapy techniques seems valuable in this disease site.

Recently, great progress has been made in the utilization of systemic therapy with the advent of immunotherapy. The PACIFIC trial demonstrated remarkable improvements in overall survival and progression free survival with the addition of durvalumab following definitive chemoradiation therapy (2). As systemic therapy continues to improve, durable locoregional control will become all the more important. It has been previously demonstrated local progression can occur in these cases up to 20 to 50% of the time (3). As such, a major challenge is to escalate radiation dose, particularly to the primary, without untoward increases in toxicity. There is literature describing the use of an SBRT boost to the primary in the locally advanced setting though this too has been limited by toxicity (4).

Each locally advanced case is unique in the geometric distribution of gross disease that is involved, particularly with respect to the mediastinum. It is not unusual to see a primary lesion at some distance from the involved mediastinal lymphatics. This primary lesion may in fact be found at the very base of the lung where significant tumor motion can lead to challenges in motion management. As a consequence, attempting to include all gross disease within a single radiation field plan can be very challenging and lead to excess dose to the heart and lungs. Moreover, treating large swaths of the thorax with a single radiation field may not adequately taken to account the varying degrees of tumor motion relative to the geometric location of the gross disease. The utilization of SBRT in the treatment of early stage NSCLC has effectively yielded local control to these lesions of over 90% in most institutional series (5, 6). Moreover, advanced radiation imaging, planning, and motion management allow for extremely high doses of radiation to be delivered over a short period of time with high fidelity despite respiratory motion.

In the present study, we identified a high-risk cohort of patients with locally advanced and oligometastatic NSCLC distributed over a large geometric volume, precluding delivery of conventional radiotherapy in a single plan. In these cases, we hypothesize that a novel combination technique with SBRT to the primary followed by intensity modulated radiation therapy to the involved mediastinal lymph nodes (MLN) could be a suitable approach.

## 2 Materials and methods

In this analysis, we included 21 patients with inoperable locally advanced stage III or oligometastastic stage IV NSCLC, featuring extensive mediastinal lymph nodes (MLN) and a peripheral or geographically distant primary tumor that underwent definitive radiotherapy between 2014 and 2017 at a single European comprehensive cancer center. Included patients also were deemed to have a high risk for a radiation-associated pulmonary damage due to preexisting intermediate or severe pulmonary conditions (e.g. chronic obstructive pulmonary disease (COPD) or emphysema) precluding the inability to undergo conventionally fractionated VMAT to all areas of gross disease as well as the lymphatic bridging region. Exclusion criteria were the inability to undergo SBRT+MLN for any reason (e.g. inability to lie still on the treatment table for a few minutes or unresolved toxic effects that influence study results). All patients were staged using a FDG-PET-CT scan and bronchoscopic lymph node biopsies. Following definitive concurrent chemoradiation, follow-up visits every three months were conducted. As treatment was delivered in an era before consolidation immunotherapy, none of our patients received durvalumab. If oligometastases were present, they were singular, outside the chest, and treated separately with local therapies (e.g. SBRT to a singular bone metastasis).

All patients were treated with SBRT and MLN treatment plans, and also had comparative VMAT only comparison plans created for dosimetric review. During the clinical evaluation, the SBRT+MLN approach was chosen given the VMAT only approach would have necessitated reductions in overall prescribed dose due to excessively large target volumes in the context of pre-existing medical comorbidities. The VMAT only treatment plans included both the primary tumor and the mediastinal lymph nodes, as well as intrapulmonary path of lymphatic drainage in a single target volume. The SBRT+MLN approach delivered hypofractionated SBRT to the primary and conventionally fractionated VMAT to the mediastinum. A four-dimensional planning CT including breath hold was performed in treatment position for each patient. Patients were immobilized in a vacuum mat. Abdominal compression was utilized as a motion mitigation device.

**Figure 1:**
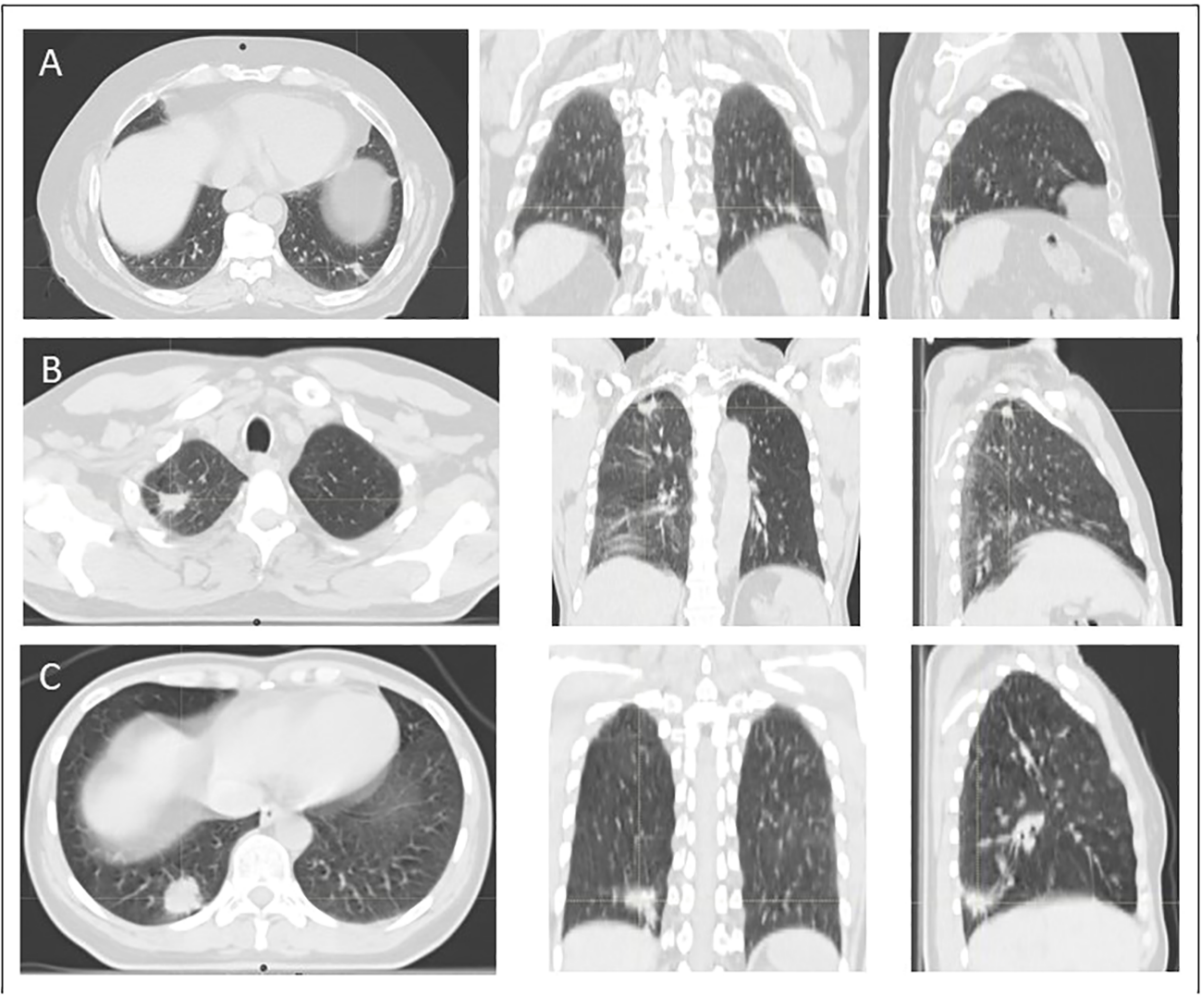
Illustration of peripheral localization of tumors included in this study. In case **(A)**, 66-year old man with a peripherally located advanced adenocarcinoma NSCLC stage III in the left lower lobe is demonstrated. Case **(B)** shows a 53-year old man with a peripherally located advanced squamous cell carcinoma NSCLC stage III in the right upper lobe. In case **(C)** a 45-year old women with a peripherally located advanced adenocarcinoma NSCLC stage III in the lower upper lobe is demonstrated. All of these patients underwent definitive concurrent platinum-based chemoradiation.

For SBRT, three-dimensional radiotherapy at a stereotaxia-adapted linear accelerator with eight multi-leaf collimated 6 megavolt photon fields was used. Primary localization in all breathing phases was used to create an internal target volume (ITV) and expanded by a safety margin of 1-2mm for clinical target volume (CTV). The CTV was further expanded with 3-5mm to take technical uncertainties into consideration, resulting in the planning target volume (PTV). The SBRT was delivered before the start of chemotherapy or during intervals between chemotherapy cycles. For MLN and VMAT only, intensity-modulated radiotherapy with 6 megavolt volumetric modulated arcs was used. A safety margin for microscopic disease of 5-6 mm in any direction was added to the gross tumor volume (GTV) for CTV creation. Finally, CTV was expanded with 6-9mm for technical uncertainties to create the PTV. Every MLN radiation therapy plan in this study included all involved lymph nodes, as well as region 7, ipsilateral region 4, and ipsilateral region 10, both in the VMAT only and in the SBRT+MLN approach. This concept was based on historical institutional guidelines as patients included in this study were treated between 2014 and 2017. **Figure 1** depicts a schematic illustration of the two treatment concepts of SBRT+MLN and VMAT only that are compared in this project.

The use of SBRT+MLN spares the ‘bridging area’ between primary tumor and mediastinal nodes which would have been included in the VMAT only approach. For MLN or VMAT only, 30-33 fractions and a total dose between 60 and 66Gy was prescribed. SBRT intended to administer a biologically effective dose (BED) to the primary of at least 100Gy. In all patients, image guidance using a kilovolt computer tomography was used prior to each treatment fraction. Treatment planning followed the principle of irradiation dose being as low as reasonably achievable (ALARA) without compromising PTV coverage. Normal tissue constraints according to QUANTEC and Emami et al. (7, 8) were used for normofractionated or AAPM TG 101 (9) for hypofractionated radiotherapy were adhered to and sometimes adapted to according to the preserved organ function e.g. lung function. Guidelines of National Comprehensive Cancer Network (NCCN) were also included.

Toxicity was evaluated using the National Cancers Institute’s CTCAE grading (version 4.03). Disease progression was assessed by reviewing follow-up imaging and toxicity assessed using all available medical records. The treatment planning CT was used as baseline and compared to the follow-up imaging studies. Tumor response was evaluated by “Response Evaluation Criteria in Solid Tumors” (RECIST) version 1.1 (10, 11). Further patient and treatment data were extracted from a clinical database maintained at our institution and from medical and official records.

The primary endpoint of the study was the lung dosimetry. Secondary endpoints included the dose exposure of the heart, esophagus and the spinal cord, as well as the clinical target volume. For the SBRT+MLN approach, EQD2 plan sums were calculated to assess overall dose exposure between both plans. Furthermore, the pneumonitis rates, the intrathoracic tumor control and overall as well as progression-free survival were assessed.

All analyses were performed following institutional guidelines and the Declaration of Helsinki of 1975 in its most recent version. Ethics approval for the study was granted by the Heidelberg University ethics committee on November 20th, 2019 (#S-767/2019). Patient confidentiality was maintained by anonymizing patient data to remove any identifying information.

Descriptive statistics for baseline variables (**Tables 1**, **2**) and for endpoints (**Tables 3**, **4**) include means (SD) and/or median (IQR and range, as appropriate) for continuous variables and absolute and relative frequencies for categorical variables. For normally distributed variables, a paired sample t test was used to test for statistical differences between data sets, otherwise the Wilcoxon signed rank test was applied. For survival analysis, Kaplan-Meier estimates were calculated. Since this is a retrospective exploratory data analysis, p-values are of descriptive nature. Statistical analyses are performed with the software R Version 4.0.3

**Table 1:** Patient baseline characteristics.

	n=21 [%]	
gender		
female	6	[28.6%]
male	15	[71.49%]
age at initial diagnosis (years)		
median	60	
minimum - maximum	44 - 77	
age at radiotherapy (years)		
median	60	
minimum - maximum	45 - 78	
prediagnosed pulmonary diseases		
overall	16	[76.2%]
COPD	7	[33.3%]
Emphysema	6	[28.6%]
Other pulmonary diseases	3	[14.3%]
prediagnosed other diseases		
overall	18	[85.7%]
cardiovascular diseases	11	[52.4%]
kidney diseases	2	[9.5%]
other diseases	5	[23.8%]
Karnosfky performance status		
median	70	
minimum - maximum	50 - 90	
smoking (pack years)		
median	45	
minimum - maximum	0 - 150	
histology		
squamous cell carcinoma	5	[23.8%]
adenocarcinoma	16	[76.2%]
UICC tumor staging		
IIIa	5	[23.8%]
IIIb	8	[38.1%]
IV	8	[38.1%]
PD-L1 expression		n=10 [%]
0%	1	[12.5%]
1-20%	4	[50.0%]
>20%	3	[37.5%]
≥50%	2	[25.0%]
driver mutations and other specific mutations		
any driver mutataions	8	[38.1%]
MAP2k	1	[4.8%]
P53	2	[9.6%]
KRAS	4	[19.1%]
U2AF1	1	[4.8%]
CDKN2A	1	[4.8%]
EGFR	1	[4.8%]
EML4-ALK	1	[4.8%]
FGFR	1	[4.8%]
BRAF	1	[4.8%]
RET	1	[4.8%]
Systemic treatment		
Chemotherapy	18	[85.7%]
Cisplatin + Vinorelbine	6	[28.6%]
Cisplatin + Gemcitabine	1	[4.8%]
Carboplatin + Etoposid	1	[4.8%]
Carboplatin + Vinorelbine	6	[28.6%]
Carboplatin + Permetrexed	3	[14.3%]
Carboplatin + Paclitaxel	1	[4.8%]

**Table 2:** Dosimetric summation comparison between combined pulmonary Stereotactic Radiotherapy (SBRT) and Volumetric Arc Therapy (VMAT) to the mediastinal lymph nodes (MLN), the SBRT+MLN approach, and both pulmonary and mediastinal VMAT, the VMAT only approach.

	VMAT only	Pulmonary SBRT	MLN VMAT	p-value for difference
	Median (Q1-Q3)	Median (Q1-Q3)		
Applied dose				
Total dose [Gy]	60.0	45.0	60.0	n.a.
Dose per fraction [Gy]	2.0	15.0	2.0	n.a.
Number of fractions	30.0	3.0	30.0	n.a.
BED [Gy]	72.0	112.5	120.0	n.a.
EQD2 [Gy]	60.0	93.8	60.0	n.a.
Target volume				
PTV dose coverage [%]	87 (85–90)	100 (100–100)	98 (85-96)	**<0.0001**
CTV Volume [ml]	441 (336-521)	307 (256-551)		**<0.0001**
Lungs				
V5 [%]	77	71		**<0.0001**
V20 [%]	36	37		0.9
Mean dose [Gy]	19.1	18.3		0.3
Heart				
D2 [Gy]	41.4	41.7		0.66
V5 [%]	49	41		**<0.0001**
V20 [%]	19	11		0.1
V25 [%]	14	9		0.5
V50 [%]	1	1		0.9
Mean dose [Gy]	12.0	8.4		0.2
Oesophagus				
V30 [%]	54	54		**0.04**
Mean dose [Gy]	32.8	34.2		0.1
Spinal cord				
Maximum dose [Gy]	40.7	40.9		0.8

For SBRT and MLN sum plans were calculated based on EQD2 for better comparison of dose on organs at risk with the VMAT only approach. For BED, α/β for tumor was assumed to be 10.

Q1-Q3, Quartile 1 - Quartile 3; Gy, Gray; BED, Biological effective dose; EQD2, Equivalent dose in 2Gy fractions; PTV, Planning target volume; CTV, Clinical target volume; V5, Volume receiving 5Gy; D2, Dose administered to 5% of organ volume.

P-values <0.05 are printed in bold.

n.a., not applicable.

**Table 3:** Pulmonary function and pneumonitis following radiotherapy.

A)	RT start	3 months after RT	6 months after RT	9 months after RT	12 months after RT		p for difference
**FEV1 [L]**	2.0 (0.8-3.6)	2.1 (0.8-3.7)	2.0 (0.7-3.2)	2.0 (0.8-3.2)	1.9 (0.8-3.0)		0.03
**FEV1 [% predicted]**	78.1 (28.9-110.1)	76.1 (28.3-110.3)	66.6 (25.4-116.9)	68.0 (27.1-94.6)	63.9 (29.1-128.9)		0.05
**FEV1/VC [%]**	69.5 (29.2-77.7)	65.0 (36.7-88.2)	65.5 (29.2-86.6)	62.8 (26.4-88.7)	62.9 (33.2-82.6)		0.8
**FEV1/VC [% predicted]**	85.1 (101.8)	84.4 (47.8-118.7)	83.5 (38.1-106.1)	80.9 (34.4-119.4)	80.9 (43.2-111.2)		0.5

**B)**				**n=21 [%]**			
Pneumonitis							
yes				2		[9.5%]	
no				19		[90.5%]	
Pneumonitis severity							
CTCAE grade I				1		[4.8%]	
CTCAE grade II				0		[0%]	
CTCAE grade III				1		[4.8%]	
CTCAE grade ≥IV				0		[0%]	
Esophagitis							
yes				13		[61.9%]	
no				8		[38.1%]	
Esophagitis severity							
CTCAE grade I				6		[28.6%]	
CTCAE grade II				5		[23.8%]	
CTCAE grade III				2		[9.5%]	
CTCAE grade ≥IV				0		[0%]	

As patients were at high risk for worsening of pulmonary function, patients were closely monitored. Pulmonary function tests were available for all patients (n=21) at all shown timepoints. Values are shown as median (min. - max.). P for difference is calculated ‘RT start vs. 12 months after RT’. P-values <0.05 are printed in bold.

RT, radiation therapy; FEV1, Forced expiratory volume in 1 second; VC, Vital capacity.

CTCAE, Common Terminology Criteria for Adverse Events.

**Table 4:** Progression following radiotherapy during follow-up.

	Overall cohort n=21 [%]		Stage III only (n=13)	Oligometastatic stage IV only (n=8)
Survival data				
death during follow-up	13	[61.9%]	7 [53.8%]	6 [75.0%]
median follow-up period in years (Q1-Q3)	2.9	(0.2 - 5.8)	3.4 (2.7 - 5.2)	2.0 (0.2 - 5.8)
Progression data*	n=20 [%]		n=12 [%]	n=8 [%]
no progression	6	[30.0%]	2 [16.7%]	4 [50.0%]
any progression	14	[70.0%]	10 [83.3%]	4 [50.0%]
primary tumor	3	[21.4%]	2 [20.0%]	1 [25.0%]
locoregional lymph nodes	4	[28.6%]	3 [30.0%]	1 [25.0%]
in-field	2	[50.0%]	1 [33.3%]	1 [100%]
out-field	2	[50.0%]	2 [66.7%]	0 [0%]
distant metastases	14	[100.0%]	10 [100%]	4 [100%]
spared tissue connection**	0	[0%]	0 [0%]	0 [0%]
lung	6	[42.9%]	5 [62.5%]	1 [25.0%]
liver	4	[28.6%]	2 [25.0%]	2 [50.0%]
brain	5	[35.7%]	4 [50.0%]	1 [25.0%]
splen	1	[7.1%]	0 [0%]	1 [25.0%]
bone	5	[35.7%]	3 [37.5%]	2 [50.0%]
adrenal gland	3	[21.4%]	2 [25.0%]	1 [25.0%]
pleura	2	[14.3%]	1 [12.5%]	1 [25.0%]
other	5	[35.7%]	3 [37.5%]	2 [50.0%]
median time to any progression in months (Q1-Q3)	10.5 (5.0-37.4)		14.0 (8-37.4)	6.0 (5.0-11.6)
median time to any intrathoracal progression in months (Q1-Q3)	23.0 (9.5-38.9)		34.9 (14.0-38.9)	8.7 (4.7-18.8)

*In one patient no sufficient imaging data were available.**Tissue connection would have been only covered with a conventionally fractionated VMAT plan but not by the combined approach of pulmonary SBRT and mediastinal conventionally fractionated VMAT in this toxicity-vulnerable cohort of advanced stage NSCLC.

## 3 Results

### 3.1 Patient characteristics

Our patient cohort was characterized by a high rate of prediagnosed pulmonary diseases and pulmonary risk factors as a consequence of longstanding tobacco abuse. The predominant NSCLC histology was adenocarcinoma in 76% (n=16). The majority of patients received definitive chemoradiotherapy in 86% (n=18) with the remainder receiving radiation therapy only. In those three patients, chemotherapy was contraindicated due to kidney failure. The vulnerable cohort presented with at least one severe organ system comorbidity and an impaired general state of health (Karnofsky performance status in median 70%). A prediagnosed pulmonary condition was present in 76% of all patients, while 86% of all patients suffered from at least one non-pulmonary prediagnosed organ system comorbidity, mainly cardiovascular diseases. Oligometastatic disease was present in 38% of patients (n=8), with the solitary site of extrathoracic metastatic disease treated with definitive local therapy. This resulted in six cases that received definitive SBRT for a singular bone metastasis and two cases that received stereotactic radiosurgery (SRS) for singular brain metastasis. In all patients, molecular data on driver mutations was available. Detailed patient baseline characteristics are shown in **Table 1**.

### 3.2 Dosimetric comparison

**Figure 2:**
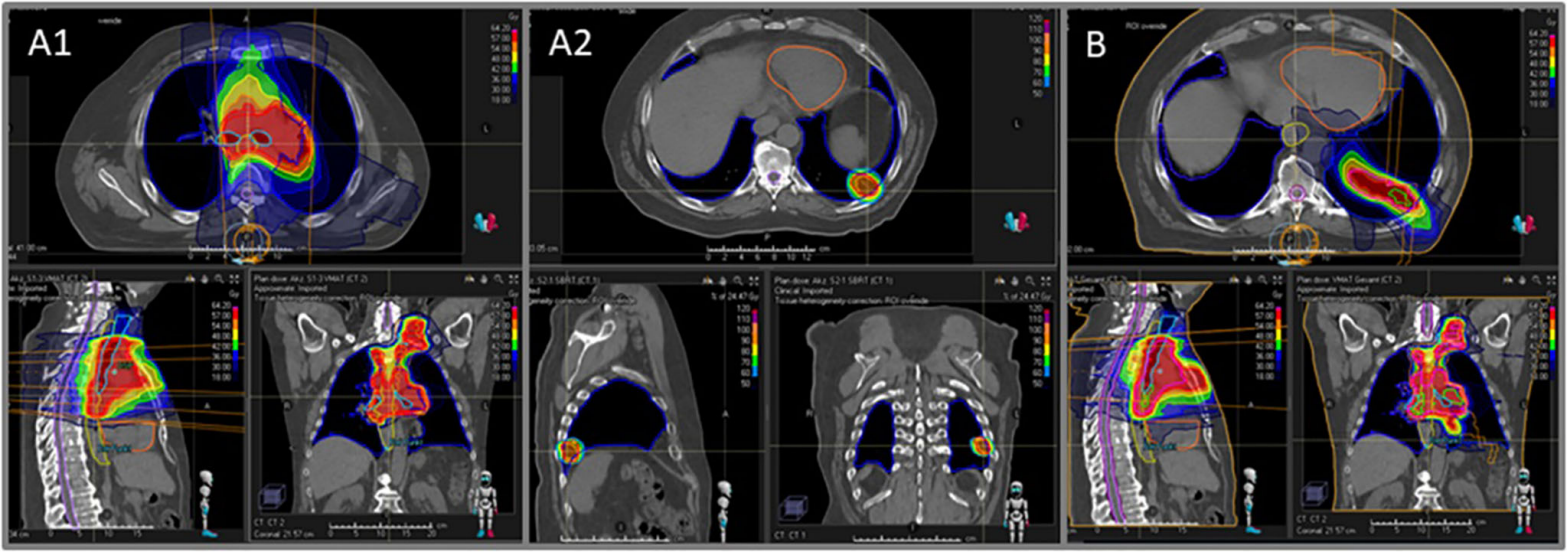
Illustration of dose color wash plans comparing conventionally fractionated radiotherapy to the mediastinal lymph nodes (MLN, **A1**) and stereotactic radio-therapy to the primary (SBRT, **A2**) (**A1**+**A2**: SBRT+MLN approach) versus conventionally fractionated VMAT for both the primary and MLN including the ‘bridging area’ (**B**: VMAT only approach). This is an exemplary case of a 62-year old man with locally advanced adenocarcinoma NSCLC stage III that underwent definitive concurrent platinum-based chemoradiation.

PTV coverage was excellent with conventionally fractionated mediastinal radiotherapy (98%) and SBRT to the primary (100%), while PTV coverage was significantly lower with VMAT only (87%; p for difference <0.0001). Furthermore, the CTV volume was significantly smaller with SBRT+MLN than with the VMAT only approach (median Volume: 307ml vs. 441ml, p<0.0001). Volume of ‘bridging area’ was in median (Q1-Q3) 34ml (13ml - 72ml). Dose constraints for organs at risk during the planning process were met in all patients. Low-dose lung exposure (median V5Gy [%]: 71 vs. 77, p<0.0001), low-dose heart exposure (median V5Gy [%]: 41 vs. 49, p<0.0001), and esophageal dose exposure (median V30Gy [%]: 54% vs. 55%, p=0.03) were all significantly reduced with the use of SBRT+MLN. Of note, there was no significant difference observed in high-dose exposure of lungs, heart, and spinal cord. Detailed dosimetric characteristics of both treatment approaches are demonstrated in **Table 2**. An example case of a 62-year old man with locally advanced adenocarcinoma NSCLC stage III that underwent definitive concurrent platinum-based chemoradiation is illustrated in **Figure 2**.

### 3.3 Pneumonitis

Following radiation therapy using SBRT+MLN, no relevant decline in pulmonary function was observed in our toxicity-vulnerable cohort. A small but significant decline of forced expiratory volume in one second (FEV1) after one year following radiation therapy was found. Pulmonary function data were available at all time points for the entire cohort (n=21, 100%) and can be seen in **Table 3A**. An overall pneumonitis rate of 10% (n=2) following SBRT+MLN was observed (**Table 3B**). In one of these two patients, pneumonitis was classified CTCAE grade 1. In this patient, pneumonitis resolved spontaneously without any intervention. In the second patient, pneumonitis was classified CTCAE grade 3 given the patient required inpatient management including corticosteroids and antibiotics. At the end of the follow-up period, this patient was alive with stable post-pneumonitis consolidation. None of our patients received immunotherapy given the era of treatment delivery, thus allowing for an evaluation of lung function and pneumonitis rates independent of immunotherapy which is rarely possible in the modern era. Detailed pneumonitis data are shown in **Table 3**.

### 3.4 Oncologic outcomes

In two patients, oncologic outcome data were not sufficient and included the following reasons (1): complete follow-up data are provided but radiotherapy was prematurely stopped after a total dose of 20 Gy due to pneumonia and was never completed due to a prolonged and severe infectious course, and (2) patient was immediately after radiotherapy lost to follow up. In 70% (n=14) of patients a tumor progression was observed during the follow-up period with a median time to disease progression of 2.9 [Q1-Q3 1.0-4.1] years. In all cases of progressive disease, the patient was found to have failed distantly. In minority of patients, (n = 2, 15%) of patients with progressive disease, failure at the site of the primary tumor was observed during the follow-up period. In 43% of patients (n=6) with progressive disease, distant pulmonary metastases occurred but all outside the ‘bridging area’ between pulmonary SBRT and mediastinal VMAT. Detailed data on disease progression are shown in **Table 4**.

**Figure 3:**
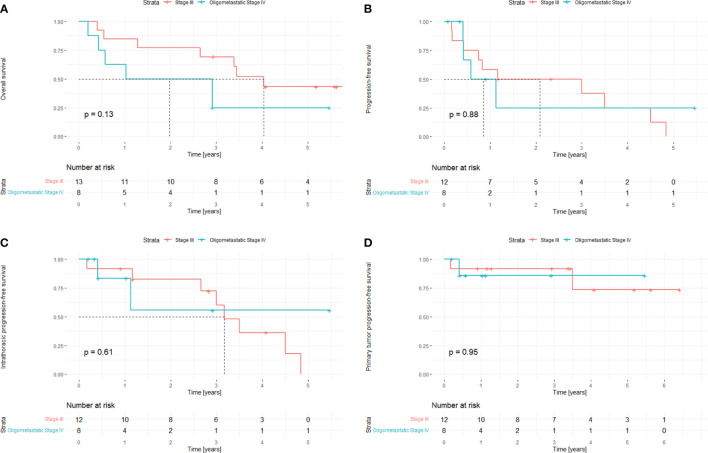
**(A–D)**. Oncologic outcomes stratified by tumor stage following the delivery of SBRT to the primary lesion and VMAT to the draining mediastinal lymph nodes: Overall survival **(A)**, progression-free survival **(B)**, intrathoracic progression-free survival **(C)** and primary tumor progression-free survival **(D)**. In one case, radiotherapy was preliminary stopped due to pneumonia and in one case follow-up data were incomplete.

Kaplan-Meier curves (**Figures 3A–D**) demonstrate overall tumor progression was primarily driven by distant metastases, not local failure at the site of the primary tumor. One- and five-year overall survival in the entire cohort was 76% and 35% (95% CIs 60%-97% and 19%-65%), respectively. Progression-free survival after one and five years was 56% and 9% (95% CIs 37%-84% and 1%-54%), respectively. One- and five-year intrathoracic progression free survival 89% and 13% (95% CIs 77%-100% and 2%-75%), respectively, with location of progression being primarily out-of-field pulmonary metastases. Primary tumor progression-free survival after one- and five years was 90% and 75% (95% CIs 77%-100% and 51%-100%), respectively. There was no significant difference between stage III and oligometastatic stage IV with additional SBRT to the single oligometastatic disease site (all p>0.1).

## 4 Discussion

The project demonstrates that SBRT+MLN could be a suitable approach for high-risk patients when geographically distant gross disease deemed unsuitable for conventional radiotherapy with VMAT only is present, as hypothesized. This approach can achieve a more favorable target volume coverage and dose distribution in the surrounding lung tissue compared to VMAT alone with low toxicity rates and without evidence for increased tumor progression rates in the spared ‘bridging area’. Prior publications have demonstrated VMAT technique to better spare healthy tissue than IMRT as VMAT achieves highly conformal dose distributions to the target (12). Nevertheless, even using the VMAT technique reaches its limits in patients with peripherally located primary necessitating large target volumes, which is particularly dangerous in patients with pre-existing pulmonary conditions, limiting dose tolerability of the healthy tissue. Given this high-risk cohort frequently suffers from pre-existing pulmonary disease, the use of VMAT alone may lead to excess treatment-related toxicity.

Even if our high-risk cohort could be thought to have a severely impaired prognosis, the presented data indicate a relatively good outcome: We compared our NSCLC stage III and oligometastatic IV sub cohort following SBRT+MLN to the PACIFIC trial NSCLC stage III sub cohort without durvalumab consolidation (placebo group) (13). While median overall survival in the placebo group of the NSCLC stage III PACIFIC trial was reached after only 2.4 years, median overall survival in our SBRT+MLN NSCLC mixed stage III and oligometastatic IV cohort was 3.4 years. It must be mentioned that patients in the PACIFIC trial were treated normofractionally with 60-66Gy in 30-33 fractions, while our SBRT+MLN approach provided a hypofractionated treatment regimen for the primary with 45Gy in 15 fractions which results in a higher biological equivalence dose and therefore higher radiobiological response. So, for the primary site, we would expect an even better tumor control with SBRT+MLN than normofractionated VMAT only.

The major concern with SBRT+MLN is whether this tissue-sparing approach might endanger intrathoracic tumor control by sparing the ‘bridging area’ between primary tumor and involved mediational lymph nodes. A randomized controlled trial would be needed to definitively answer on that question. Nevertheless, our retrospective follow-up data of the treated patients did not point to such concerns. Not a single patient in our cohort was affected by a tumor recurrence within the spared ‘bridging area’. We know from recurrence pattern analyses in literature for early stage NSCLC that underwent SBRT that distant tumor recurrences are with about two thirds the most frequent site of failure (14). It is already well known that limiting the lymphatic path irradiation to an involved field approach is well suitable to reduce toxicity without considerably endangering intrathoracic tumor control as only 6% of tumor recurrences occur in elective lymph node areas (15). Our proposed tissue-sparing approach includes all involved fields but waives the ‘bridging area’ between primary and mediastinum. The approach of including the bridging area originates from an era before ESTRO ACROP guideline from 2018 (16) and when no evidence existed that ‘bridging area’ can be safely spared. At that time, no standardized international way of CTV delineation existed for locally advanced NSCLC. As dose can be escalated within the involved field by sparing irradiated healthy tissue, tumor control probability was even increased according to an in-silico trial (17). To sum up, even if the statistical power of our results is limited due to the small number of patients, our data demonstrate that concerns about an insufficient radiation therapy with SBRT+MLN can be reassured.

The incremental influence of toxicity as a limiting factor in dose-escalation trials was clearly demonstrated by the RTOG0617 trial. In this trial 544 patients were randomized to 74 Gy versus 60 Gy given in 2 Gy fractions with concurrent chemotherapy for patients with stage III non-small-cell lung cancer. Ultimately, the results demonstrated significantly worse outcomes in the high dose arm. Hence, the study group even concluded that such a dose escalation may be harmful as they observed more treatment-related deaths in the high-dose chemoradiotherapy group than in the low-dose chemoradiotherapy group (eight vs three patients) (1).

In our cohort, we observed a overall pneumonitis rate <10% and a severe pneumonitis rate (CTCAE ≥°3) of <5% with no proof of relevant decline in pulmonary function. Literature reports that pre-SBRT pulmonary function does correlate with overall survival but nor cause-specific survival (18). Also, in cases of small target volume, SBRT was even able to improve pulmonary function (18). Interestingly, literature data discuss that rate of severe pneumonitis was independent of pre-SBRT pulmonary function and found a pneumonitis CTCAE ≥°2 rate of 7% in a cohort of early-stage NSCLC where SBRT is standard treatment if surgery is rejected (18, 19). Especially in the light of an additional low-dose lung exposure due to MLN combined with SBRT to the primary, pneumonitis rate in our cohort is very favorable. Good clinical outcome is supported by the dosimetric comparison that demonstrated that low-dose lung, heart and esophagus dose exposure were all significantly reduced with SBRT+MLN. Especially the heart is suspected to be vulnerable to even low-dose exposure since but not limited to RTOG 0617.

An important point that needs to be discussed with the results of the RTOG 0617 trial is that treatment planning was not based on a diagnostic PET scan in most patients. The resulting stage migration might be one reason why the low-dose radiotherapy was more effective than expected. To reduce irradiation doses to organs at risk, the involved field approach was demonstrated to be a sufficient (15, 20). The PET-boost trial demonstrated that PET-based dose escalation to the primary tumor or high FDG uptake regions was possible in three quarters of patients whilst maintaining pre-defined dose constraints (21). The PET-plan trial investigated imaging-based target volume reduction in chemoradiotherapy for locally advanced NSCLC. While no increase in toxicity was observed, 18F-FDG PET-based planning could potentially improve local control (22). This trial demonstrates that a PET-based reduction of target volume is safely achievable. Moreover, no increase in tumor recurrences was observed, locoregional tumor control was even superior in the PET-based target group than in the conventional target group. Combining PET-based treatment planning with the SBRT+MLN approach might further improve results for toxicity-vulnerable cases of advanced stage NSCLC.

Given the results of the aforementioned trials, sparing the ‘bridging area’ with our SBRT+MLN approach could be a pragmatic way to both reduce normal tissue toxicity and to increase tumor control by intratumoral dose escalation in patients that are unsuitable for conventional radiotherapy using VMAT only. Further investigations are warranted to determine if an 18F-FDG PET to the SBRT+MLN approach could further increase safety and efficacy in patients unsuitable for VMAT only. Limitations for this analysis were the dosimetric character of the comparison and the relatively small cohort. Changes of guidelines and literature data during the follow-up period of a perennial study is a well-known problem that also affects the presented data. Furthermore, target volume definition in locally advanced NSCLC was not internationally homogenous in the past years. Nevertheless, the analysis was not only dosimetric, but also a clinical follow-up for the patients following SBRT+MLN. These data firstly evaluated dosimetrically and clinically a potential approach for patients that are unsuitable for the conventional VMAT only approach due to intolerable dose on adjacent lung tissue. In the next step it would be interesting to better understand which patients should be treated with which approach. Even if an available PET scan is nicely enhancing treatment planning, unfortunately PET scans are by far not available for all patients in clinical practice. So, we should better understand based on clinical risk factors which patients could profit the most from which approach. Based on our personal impressions gained with the presented analysis, we would regard this approach to be suitable at least for high risk patients with an impaired general state of health (Kanofsky index ≤70%), significant prediagnosed diseases of organ systems that would be affected by low-dose exposure (lungs, primary close to the predamaged organ e.g. heart, kidney or liver), peripherally located primary and cases that would necessitate dose reductions with the conventional VMAT only approach due to expected high toxicity risks. Whether other patients might also profit from this approach needs to be further investigated. As SBRT+MLN goes along with a dose escalation at the primary site while sparing tissue it is worth to further investigate that question for other patients.

## 5 Conclusions

In high-risk patients with peripherally located primary and impaired pulmonary dose tolerance due to pre-existing pulmonary conditions or other dose-limiting situations, SBRT+MLN is suitable and in fact favorable to VMAT only treatment. No tumor recurrences in the spared ‘bridging area’ were observed. This approach can limit toxicity and provide an alternative to dose reductions necessitated by otherwise very large target volumes or otherwise failed dose constraints at organs at risk.

## Data availability statement

The datasets analyzed for this study will not be made publicly available since national legislation and the terms of study ethics approval do not allow dataset sharing outside of the institutions participating in the analysis.

## Ethics statement

All analyses were performed following institutional guidelines and the Declaration of Helsinki of 1975 in its most recent version. Ethics approval for the study was granted by the Heidelberg University ethics committee on November 20th, 2019 (#S-767/2019). Patient confidentiality was maintained by anonymizing patient data to remove any identifying information. No written informed consent was needed.

## Author contributions

TE, SRi, JD and RS planned and supervised this analysis. TE, CS, ET-M performed data extraction and analysis. TE and RS performed statistical analysis. TE reviewed data analysis and TE and JL drafted the manuscript. All authors contributed data and participated in reviewing and improving analysis and manuscript. All authors contributed to the article and approved the submitted version.

## Supplementary material

Supplementary Figure 1 Schematic Illustration of the two treatment techniques. **(A)** Depicts the SBRT+MLN approach with SBRT to the primary being illustrated in red and MLN irradiation being illustrated in orange. **(B)** shows the VMAT only approach illustrated in orange. Chemotherapy was not given during the SBRT portion of the treatment.
