# Supplementary figures and images for: High-risk patients with locally advanced non-small cell lung cancer treated with stereotactic body radiation therapy to the peripheral primary combined with conventionally fractionated volumetric arc therapy to the mediastinal lymph nodes
Source: Front Oncol. 2023 Jan 13;12:1035370. doi: 10.3389/fonc.2022.1035370 (PMC9880536; doi:10.3389/fonc.2022.1035370)

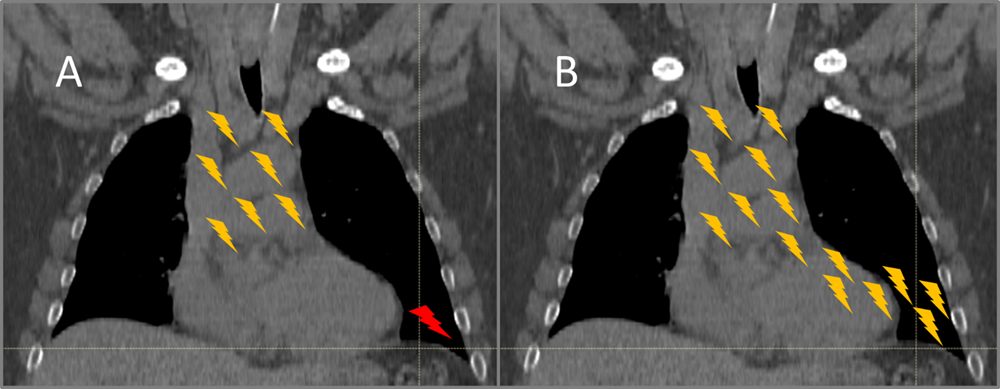

Supplement: Supplementary Figure 1 — Schematic Illustration of the two treatment techniques. (A) Depicts the SBRT+MLN approach with SBRT to the primary being illustrated in red and MLN irradiation being illustrated in orange. (B) shows the VMAT only approach illustrated in orange. Chemotherapy was not given during the SBRT portion of the treatment. [file Image_1.jpeg]
